# Ion Homeostasis and Metabolome Analysis of *Arabidopsis* 14-3-3 Quadruple Mutants to Salt Stress

**DOI:** 10.3389/fpls.2021.697324

**Published:** 2021-09-13

**Authors:** Jing Gao, Paula J. M. van Kleeff, Mark H. de Boer, Alexander Erban, Joachim Kopka, Dirk K. Hincha, Albertus H. de Boer

**Affiliations:** ^1^Institute of Apicultural Research, Chinese Academy of Agricultural Sciences, Beijing, China; ^2^Department of Structural Biology, Faculty of Earth and Life Sciences, Vrije Universiteit Amsterdam, Amsterdam, Netherlands; ^3^Department of Plant Physiology, Swammerdam Institute for Life Sciences, University of Amsterdam, Amsterdam, Netherlands; ^4^Department Willmitzer, Max Planck Institute Molecular Plant Physiology, Potsdam, Germany; ^5^Department of Medicinal Chemistry, Beta Faculty, Vrije Universiteit Amsterdam, Amsterdam, Netherlands

**Keywords:** 14-3-3, salinity, metabolism, ion homeostasis, plant abiotic stress

## Abstract

Salinity is one of the major abiotic stresses that limits agricultural productivity worldwide. Many proteins with defined functions in salt stress adaptation are controlled through interactions with members of the 14-3-3 family. In the present study, we generated three 14-3-3 quadruple knockout mutants (qKOs: *klpc, klun*, and *unpc*) to study the role of six non-epsilon group 14-3-3 proteins for salt stress adaptation. The relative growth inhibition under 100 mM of NaCl stress was the same for wild-type (Wt) and qKOs, but the accumulation of Na^+^ in the shoots of *klpc* was significantly lower than that in Wt. This difference correlated with the higher expression of the *HKT1* gene in *klpc*. Considering the regulatory role of 14-3-3 proteins in metabolism and the effect of salt stress on metabolite accumulation, we analyzed the effect of a 24-h salt treatment on the root metabolome of nutrient solution-grown genotypes. The results indicated that the *klpc* mutant had metabolome responses that were different from those of Wt. Notably, the reducing sugars, glucose and fructose, were lower in *klpc* under control and salt stress. On the other hand, their phosphorylated forms, glucose-6P and fructose-6P, were lower under salt stress as compared to Wt. This study provided insight into the functions of the 14-3-3 proteins from non-epsilon group members. In summary, it was found that these proteins control ion homeostasis and metabolite composition under salt stress conditions and non-stressed conditions. The analyses of single, double, and triple mutants that modify subsets from the most effective qKO mutant (*klpc*) may also reveal the potential redundancy for the observed phenotypes.

## Introduction

Salinity is one of the major abiotic stresses that limits agricultural productivity worldwide. Drought periods, in combination with irrigation, results in an increase in the secondary salinization of agronomically important soils and a loss of crop yield (Fita et al., 2015). At the cellular level, osmotic stress and ion toxicity are the two sides of salt stress that limit growth and, in turn, productivity (Yadav et al., 2011). During the first phase of salt stress, plant growth is inhibited by the osmotic effect, which reduces water uptake and turgor pressure. The second phase then relates to ion over-accumulation resulting in cell death (Munns and Tester, 2008). As a consequence of these two phases, secondary stresses, such as nutritional imbalance, oxidative stress, and infections, are also induced by salt stress (Isayenkov, 2012). Subsequently, the metabolic composition of the plants changes (Richter et al., 2015).

In order to adapt and survive under high-salinity conditions, a signaling network, including various ion transporters, signaling molecules [e.g., Ca^2+^, abscisic acid (ABA)], transcription factors, protein kinases, organic osmolytes, reactive oxygen species, etc., are triggered when plants are exposed to high salinity (Golldack et al., 2014). For example, SOS3 encodes a calcium sensor and activates SOS2 protein kinase activity in a Ca^2+^-dependent manner (Halfter et al., 2000). A calcium activated SOS3/SOS2 complex then maintains the cellular Na^+^ homeostasis through phosphorylation and the activation of the Na^+^/H^+^ antiporter SOS1 (Qiu et al., 2002; Quintero et al., 2002). In addition, the SOS3-like Ca2^+^-binding protein/calcineurin B-like protein (SCaBP8/CBLs) regulates the degradation of 14-3-3 proteins in response to salt stress (Tan et al., 2016). During this salt stress, the phosphorylation and interaction between SOS2-like protein kinase 5 (PKS5) and SOS2 could increase the interaction between SOS2 and *Arabidopsis* 14-3-3 kappa and lambda, which further repress SOS2 activity (Yang et al., 2019). These findings suggest that 14-3-3 and SOS3 proteins coordinately activate/inactivate the downstream protein kinases SOS2 and PKS5 to regulate cellular Na^+^ homeostasis. Furthermore, the overexpression of the plasma membrane transporter SOS1 C-term leads to the sequestration of inhibitory 14-3-3 proteins, which makes SOS1 more activated and thereby improving salt tolerance in *Arabidopsis* (Duscha et al., 2020). *Arabidopsis* has a single gene that encodes the HKT1 protein, which is expressed in root stellar cells and controls the root:shoot allocation of Na^+^ by means of Na^+^ retrieval from the xylem (Xue et al., 2011). The overexpression of AtHKT1 is also conducive to the regulation of K^+^ status and reduction of Na^+^ toxicity under salt stress (Wang et al., 2018). In addition, HKT1 and the SOS-pathway are interconnected, since *HKT1* mutations suppress the Na^+^ hypersensitivity of *sos3-1* mutant seedlings (Rus et al., 2001; Wang et al., 2019). Thus, Na^+^ and K^+^ homeostasis in *Arabidopsis* under salt stress is improved through the coordinated expression of *AtHKT1;1* and *AtSOS1* (Wang et al., 2014b).

Salt stress also induces many metabolic changes as a result of an altered gene expression and the post-translational modification of proteins (Sanchez et al., 2008). In particular, the ability to maintain intracellular compatible solute pools can reduce the damage caused by high extracellular osmolality when plants suffer salt stress. Multiple metabolomics studies have also shown that the pool sizes of many metabolites that share the properties of polarity, high water-solubility and no net charge at physiological pH, such as organic acids, amino acids, sugars, polyols, and sulfonium compounds, increase under salinity stress (Alla et al., 2012; Hill et al., 2013; Wu et al., 2013; Richter et al., 2015). Nevertheless, how these global metabolic responses participate in osmotic stress, ion homeostasis, and detoxification and whether the metabolic phenotype correlates with its ability to tolerate certain levels of osmotic stress remain poorly understood.

Proteins from the 14-3-3 family are a class of molecular chaperones that have important functions in the adaptation of plants to salt and drought stress (Chen et al., 2006; Kaeodee et al., 2011). These functions are explained by the interaction of 14-3-3s with proteins that control ion homeostasis (Bunney et al., 2002; De Boer et al., 2013), ABA signaling (Sirichandra et al., 2010; Sun et al., 2015), and metabolism (Chung et al., 1999; Shin et al., 2011). The 14-3-3 protein is also one of the key regulators for coordinating many plant metabolic pathways, e.g., nitrate and carbohydrate metabolism (Lambeck et al., 2010; Gao et al., 2014; Wang et al., 2014a). Furthermore, 14-3-3 proteins do not have enzymatic activity, but act as sensors for target protein phosphorylation, whereby the phosphorylation of a specific motif dramatically increases the affinity for the target, e.g., 14-3-3 proteins bind to the phosphorylated C-terminus of H^+^-ATPases (Baunsgaard et al., 1998), whereas the phosphorylation of the C-terminal motif can be driven by light (Kinoshita and Shimazaki, 2002), auxin (Takahashi et al., 2012), and salt stress (Shan'ko and Babakov, 2002).

Since 14-3-3 proteins control so many proteins/enzymes with a function in salt stress adaption, it is to be expected that knockout mutations in 14-3-3 genes will result in salt stress-related phenotypes. However, the *Arabidopsis* genome contains 13 expressed 14-3-3 genes, with redundancy possibly existing between these isoforms (Roberts and De Bruxelles, 2002; Paul et al., 2012). To address the question of redundancy and/or specificity, we generated a range of stacked mutants in the non-epsilon group of 14-3-3 paralogs (Van Kleeff et al., 2014). The analyses of root growth, hormone sensitivity, and, more specifically, the activation of a neutral cytosolic invertase indeed showed both specificity and redundancy among the six genes analyzed (Gao et al., 2014; Van Kleeff et al., 2014). Thus, in this study, we used a set of 14-3-3 quadruple knockout mutants (14-3-3 qKOs) composed of *kappa*/*lambda*/*upsilon*/*nu* (*klun*), *kappa*/*lambda*/*phi*/*chi* (*klpc*), and *upsilon*/*nu*/*phi*/*chi* (*unpc*) to get insight in the role that higher order *Arabidopsis* 14-3-3 mutants play in ion homeostasis and metabolite production during salt stress adaptation. We determined the accumulation of Na^+^ and K^+^ in the rosette leaves and flower stalks of the 14-3-3 qKOs and Wt plants. In addition, the root metabolic profiles of the different genotypes in response to salt stress were analyzed by gas chromatography–mass spectrometry (GC-MS). Principal component analysis (PCA), orthogonal projections to latent structures discriminant analysis (OPLS-DA), and hierarchical cluster analysis (HCA) were used to show that the root metabolite composition of mutant plants differed from those in Wt roots. The expression of HKT1, which was previously identified as significantly altered in salt stress in *Arabidopsis*, was also investigated by quantitative reverse transcription PCR (qRT-PCR). Taken together, our results demonstrated that 14-3-3 proteins are involved in multiple salt stress-related metabolic and signaling pathways, with both redundancy and specificity between the 14-3-3 proteins.

## Materials and Methods

### Plant Growth Conditions

The homozygous 14-3-3 quadruple KO mutants were generated by crossing the double mutants as described in a previous study reported by Van Kleeff et al. (2014). The seeds of the homozygous *athkt1;1* mutant (Columbia-0) were obtained from the Nottingham Arabidopsis Stock Center (NASC) (N6531). These seeds [*Arabidopsis thaliana* Columbia ecotype (Col-0)] were also surface-sterilized in 70% ethanol for 10 min, washed in 25% bleach + 0.1% Tween-20 (Sigma-Aldrich, St. Louis, MO, USA) for 10 min, subsequently washed three times with sterilized MilliQ (Millipore, Burlington, MA, USA), and resuspended in 0.1% sterile agarose. The seeds were then placed onto a half-strength Murashige and Skoog (MS) medium (Murashige & Skoog, 1962; Sigma-Aldrich, Hamburg, Germany) solidified with 1% of plant agar (Sigma-Aldrich, Burlington, MA, USA) (pH 5.8) and stratified for 3 days at 4°C. For germination, plates were placed vertically in a growth chamber with 14 h of light, 170 μmol m^−2^ s^−1^ (22°C)/10 h of darkness (18°C). For the biomass and ion content measurement, the seedlings were transplanted to 8-cm pots (with one seedling per pot) filled with regular garden soil in a greenhouse 15 days after germination. Plants were then watered every 2 days. For NaCl treatment, the pots were incubated in 2-cm deep water and supplemented with 0 or 100 mM NaCl for 2 h. This was repeated twice with a 3-day interval. After 14 days, the plants were harvested, and the fresh weight of the rosette leaves and flower stalks was immediately determined. After weighing, the plant material was dried at 75°C for 2 days, with the dry material subsequently being used for Na^+^ and K^+^ content measurement.

### Na^+^ and K^+^ Content Measurement

Na^+^ and K^+^ concentrations were determined in leaves and flower stalks by high performance liquid chromatography (HPLC Shimadzu Class-LC10A system, Shimadzu Corporation, Kyoto, Japan). Then, the oven-dried plant material was ground into a powder using a mortar and pestle. After that, 5 mg of the dried material was weighed and boiled in tubes containing 5 ml of MilliQ water for 1 h at 100°C in a water bath. After boiling, samples were filtered through 0.2-mm filters (Whatman, Maidstone, United Kingdom) and Na^+^ and K^+^ concentrations were determined by injecting 5 μl of sample in a Shimadzu HPLC system (10 Series, Shimadzu) equipped with an IC-YS50 column (Shimadzu). As an eluent, 4 mM of methane sulfonic acid was used in an HPLC-grade H_2_O (J.T. Baker, Deventer, the Netherlands) with a flow rate of 1 ml/min. A calibration curve was then made by injecting 1, 2, 5, 10, and 25 μl of a standard solution containing 5 mM of NaCl and 5 mM of KCl.

### Water Loss Rate

The rate of water loss in Wt plants and 14-3-3 mutants growing in the green house was measured. These plants were grown in pots with regular garden soil for 24 days before salt treatment. Each pot was covered with aluminum foil to prevent water loss from the soil surface. The next day, the plants were placed in trays with water or 100 mM of NaCl (pots were 3 cm deep in the solution). The pots were left for 3 h to absorb the water or salt, followed by the removal of adhering water, and then the weight loss of each pot was recorded over time inside the greenhouse. After the experiment, the shoot fresh weight of each plant was determined and used to calculate the rate of water loss per unit time per unit fresh weight.

### Real-Time PCR Assay

To analyze the expression pattern of *AtHKT1* in the Wt plants and 14-3-3 mutants, quantitative RT-PCR analyses were performed. The plants used were from the same batch of plants that were used for the transpiration experiment. Here, the greenhouse plants were treated with 0 or 100 mM of NaCl for 1 day. Then, the plants were carefully pulled from the soil, with between 1 and 2 cm of the main root being harvested and the roots from three plants being pooled for each treatment. Furthermore, the soil was carefully removed and the taproots were frozen in liquid nitrogen. The total RNA was extracted from the root tissue using the NucleoSpin® RNA Plant Kit (Macherey-Nagel, Düren, Germany), and first-strand cDNA was synthesized from 2 mg of the total RNA using the Superscript II Kit (Invitrogen, Waltham, MA, USA) with oligo d(T)18 primers according to the instructions of the manufacturer. Quantitative RT-PCR reactions contained 100 ng of cDNA, 1 pmol of each primer (HKT1, forward primer 5′-3′ TATGGGTTTGCAGGACGATGGAGT, reverse primer 5′-3′ GCCAGATTTGGCTGTGAACTGCTT; Actin 2 forward primer 5′-3′ AGTGGTCGTAC AACCGGTATTGT, reverse primer 5′-3′ GATGGCATGAGGAAGAGAGAAAC; UBQ5 forward primer 5′-3′ AGGCGAAGATCCAAGACAAG, reverse primer 5′-3′ TGAACCTTTCCAGATCCATCG), and 2 × SYBR Green PCR buffer (Bio-Rad, Hercules, CA, USA). The PCR conditions for the amplification of *HKT1* and actin were as follows: 1 min at 94°C, followed by 30 cycles of 45 s at 94°C, 60 s at 54°C and 75 s at 72°C. The PCR products were examined according the 2^−ΔΔCT^ method (Livak and Schmittgen, 2001). Finally, five biological replicates and three technical replicates were analyzed.

### Metabolome Profiling

For the metabolomics analysis in the roots of the 14-3-3 qKOs, the plants were grown in a greenhouse with a one-half strength Hoagland solution, i.e., 3 mM of KNO_3_, 2 mM of Ca(NO_3_)_2_, 1 mM of NH_4_H_2_PO_4_, 20 μM of Fe-EDTA,0.5 mM of MgSO_4_, 1 μM of KCl, 25 μM of H_3_BO_3_, 2 μM of MnSO_4_, 2 μM of ZnSO_4_, and 0.1 μM of CuSO_4_,0.1 μM of (NH_4_)_6_Mo_7_O_24_ at pH 5.5. When the plants were 22 days old, the Hoagland solution was replaced by a fresh identical solution or by a one-half strength Hoagland solution supplemented with 100 mM of NaCl. After 24 h, the roots were harvested, immediately rinsed with Milli Q water for 10–20 s, dried rapidly on tissue paper, and snap frozen in liquid nitrogen. Total root systems were ground in liquid nitrogen, weighed, and stored until further processing at −80°C.

Metabolite profiling by GC-time of flight–MS (GC-TOF-MS) was performed as described in previous studies (Lisec et al., 2006; Erban et al., 2007) by split-less and subsequent split injections of the same samples (Supplementary Table 1). Approximately 50 mg of frozen ground material was then homogenized in 300 μl of methanol at 70°C for 15 min. Then, 200 μl of chloroform was added, and extraction continued at 37°C for 5 min. Next, the polar fraction was prepared by liquid partitioning through the addition of 400 μl of water. A dried 160 μl aliquot of the polar fraction was then chemically derivatized by methoxyamination and subsequent trimethylsilylation. After that, samples were analyzed using GC-TOF-MS (ChromaTOF software, Pegasus driver 1.61; LECO Instrumente GmbH, Mönchengladbach, Germany). Furthermore, the chromatograms and mass spectra were evaluated using the TagFinder (Luedemann et al., 2008) and NIST05 softwares (http://www.nist.gov/srd/mslist.cfm). Metabolite annotation was manually supervised using the mass spectral and retention index collection of the Golm Metabolome Database (Kopka et al., 2005; Hummel et al., 2010), with the annotation criteria being reported in Supplementary Table 1. The peak heights of the recorded mass fragments were normalized on the basis of the fresh weight of the sample and an internal standard added during extraction [(^13^C_6_)-sorbitol] and subsequently maximum scaled per metabolite, i.e., normalized abundances (Watanabe et al., 2013). Each condition was analyzed by 4–5 biological replicates.

### Data Analysis

The biomass and Na^+^ and K^+^ concentrations were tested using a two-way ANOVA followed by a Tukey test for multiple comparisons (*p* < 0.05) using SPSS 19.0. The normalized abundances of the annotated known yet non-identified metabolites from the root metabolite profiles were statistically assessed by ANOVAs, a Mack–Skillings test, and pairwise comparisons *via* a heteroscedastic Student's *t*-test. These calculations and heat map visualizations were performed by the Multi-Experiment Viewer software, MeV [Version 4.9.0; http://www.tm4.org/mev/ (Saeed et al., 2003, 2006)], or by Microsoft Office Excel 2016 and the SAS statistical analysis software (SAS Institute Inc., Cary, NC, USA). Then, the log_2_-transformed response ratio data were subjected to multivariate analysis by fitting PCA-X and OPLS-DA using the SIMCA-P software v.14.1 (Umetrics, Umea, Sweden). A hierarchical clustering analysis of selected metabolites was also performed by MeV (Version 4.9.0) using the replicate means of the Wt plants.

## Results

### 14-3-3 Mutants Show Reduced Na^+^ Accumulation During Salt Stress

Three 14-3-3 quadruple KOs, namely, *kappa*/*lambda*/*phi*/*chi* (*klpc*), *kappa*/*lambda*/*upsilon*/*nu* (*klun*), and *upsilon*/*nu*/*phi*/*chi* (*unpc*), were used in this study. The quadruple mutants were generated by crossing two double mutants: kl^*^pc, kl^*^un, and un^*^pc (Supplementary Figure 1A) (for details, see Van Kleeff et al., 2014). T-DNA insertions were also monitored using PCR, and the mutants were scored on full-length 14-3-3 transcripts (Supplementary Figure 1B). To check whether the 14-3-3 protein levels were altered by the loss of four 14-3-3s, Western blotting with a 14-3-3 antibody [pan 14-3-3, (K-19) (Santa Cruz Biotechnology, Santa Cruz, CA) was performed to analyze the 14-3-3 protein levels in the 14-3-3 qKOs (Supplementary Figure 1C). As a result, a loss of the upper band was observed in the *klun* mutant, suggesting that the reduction in the total 14-3-3 protein levels was consistently observed in *klun*.

To evaluate the physiological effects of high salinity on the three 14-3-3 qKOs, plants were germinated for 15 days on agar plates and then grown with garden soil in a greenhouse. The experiment plants were then exposed to 0 or 100 mM of NaCl during the last 2 weeks. Shoot fresh weight and the accumulation of Na^+^ and K^+^ in rosette leaves (L) and flower stalks (F) of the mutants were also determined and compared to those of the Wt plants. As a Na^+^ hyper-accumulator, we also included the *Athkt1* mutant in the experiment. Two independent experiments were done: one in the winter (Exp. 1: from December to January, with 18–20°C, 8-h photoperiod, and a relative humidity of 70%) and the other in the summer (Exp. 2: from July to August, with 25–28°C, 14-h photoperiod, and a relative humidity of 75%).

#### Plant Growth

**Figure 1:**
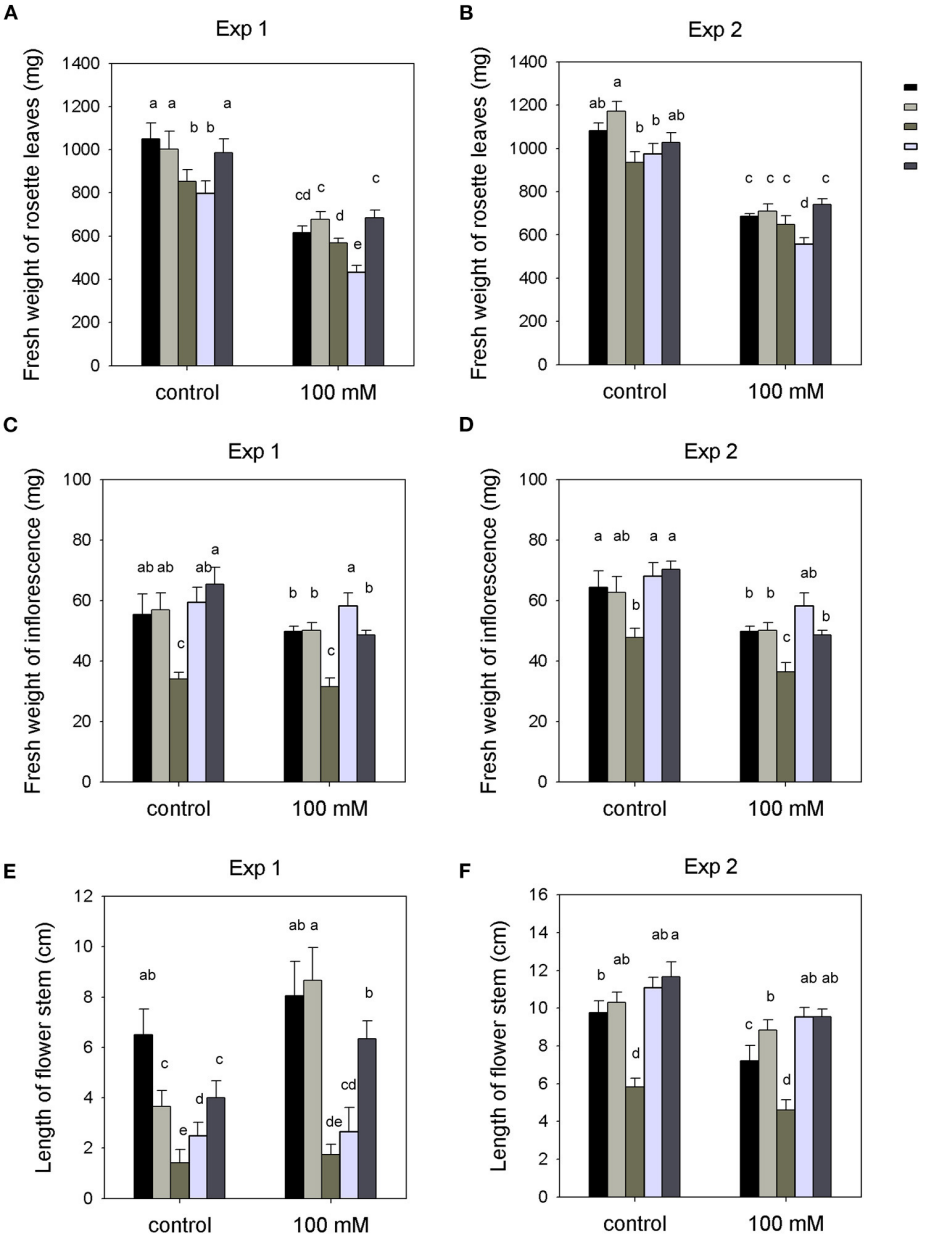
Shoot biomass production of wild-type (Wt), 14-3-3 quadruple knockout mutants (qKOs), and *hkt1* mutant plants grown in soil with and without (= control) the addition of 100 mM of NaCl to the pots. Two independent experiments were done: Exp. 1 (in the winter) and Exp. 2 (in the summer). **(A,B)** fresh weight of rosette leaves. **(C,D)** fresh weight of inflorescence. **(E,F)** length of flower stem. Values are averages ± SE (*n* = 3~5). Different letters above the column indicate significant differences between the treatments or genotypes according to a two-way ANOVA (*P* < 0.05; Tukey's test). Statistics can be found in Supplementary Table 1.

A two-way ANOVA showed a significant varietal effect of salinity on the fresh weight of rosette leaves of all experiment plants after the 100-mM NaCl treatment (*P* < 0.001), while there were no significant interactions between genotype and NaCl treatment (Figure 1 and Supplementary Table 1). Overall, the mutant plants showed the same relative growth inhibition by salt as the Wt plants (~30–40%) (Supplementary Figures 2, 3). The *unpc* mutant showed a higher reduction in rosette leaf growth with 100-mM NaCl compared to Wt (two-way ANOVA interaction, *P* < 0.001), while the other two 14-3-3 qKOs (*klpc* and *klun*) were indistinguishable from Wt (Figures 1A,B). Furthermore, the inflorescence fresh weight and flower stem length of the *klun* mutant was much lower than those of the Wt plants (Figures 1C–F). In the experiment conducted in the winter, *klpc* and *unpc* also showed shorter flower stems compared with Wt plants, but the differences were not observed in the experiment that was carried out in the summer (Figures 1E,F). Notably, the roots were not included since it was not possible to harvest all the roots of each plant from the soil.

#### Sodium and Potassium Concentrations

**Figure 2:**
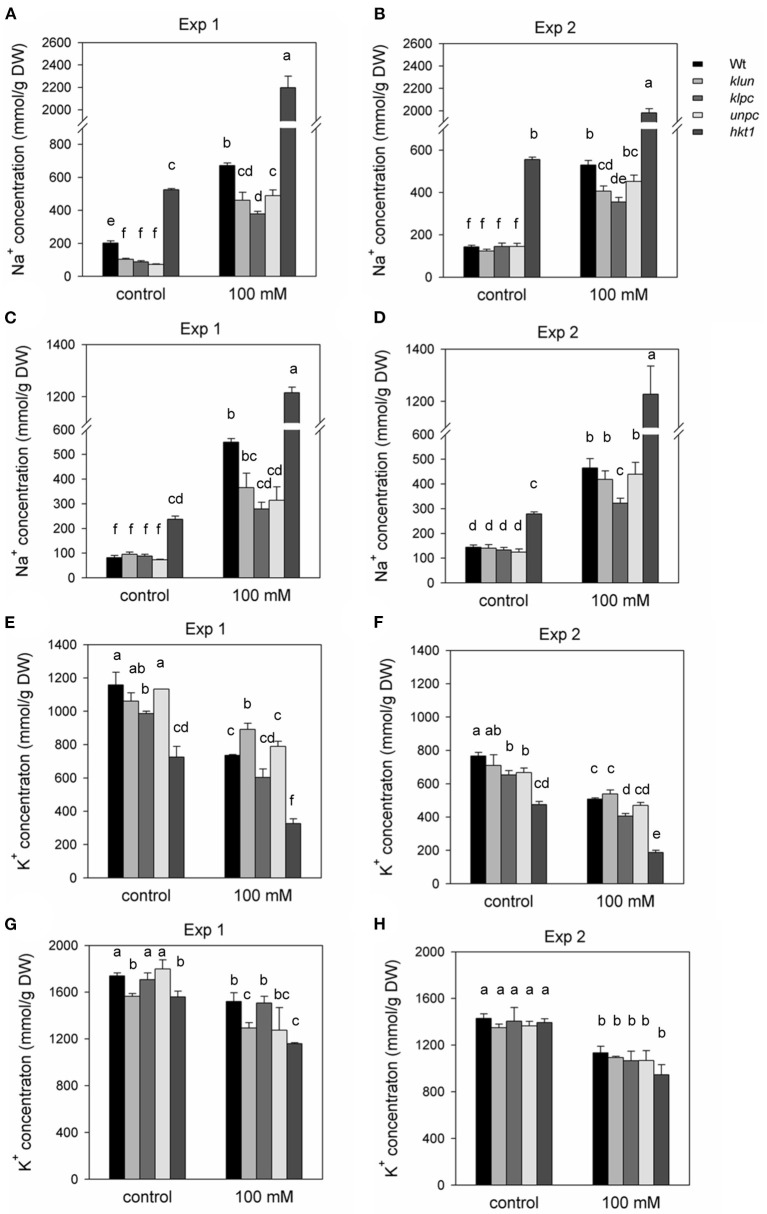
Na^+^ and K^+^ contents of the rosette leaves and flower stalks of Wt, 14-3-3 qKOs, and *hkt1* mutant plants grown in soil with and without (= control) the addition of 100 mM of NaCl. Two independent experiments were done: Exp. 1 (in the winter) and Exp. 2 (in the summer). **(A,B)** Na^+^ content of rosette leaves. **(C,D)** Na^+^ content of inflorescence. **(E,F)** K^+^ content of rosette leaves. **(G,H)** K^+^ content of inflorescence. Values are averages ± SE (*n* = 3~5). Different letters above the column indicate significant differences between the treatments or genotypes according to a two-way ANOVA (*P* < 0.05; Tukey's test). Statistics can be found in Supplementary Table 2.

Next, we evaluated salt accumulation as Na^+^ and K^+^ contents in the shoots. The amounts of Na^+^ and K^+^ in rosette leaves and flower stalks are shown in Figure 2. The Na^+^ and K^+^ contents of rosette leaves were significantly influenced by genotype and NaCl treatment, and there were significant interactions between genotype and NaCl treatment (Supplementary Table 2). The Na^+^ hyper-accumulating phenotype of *hkt1* plants as reported in other studies (Maser et al., 2002; Rus et al., 2006; Jha et al., 2010) was also evident in our study; for instance, even in plants from pots where no NaCl was added, the *hkt1* plants accumulated relatively large amounts of Na^+^ in the shoots (*P* < 0.001). This showed that, even at low soil Na^+^ levels, an efficient Na^+^-exclusion mechanism is operational in Wt and 14-3-3 mutant plants. Furthermore, in the summer experiment, all 14-3-3 mutant plants grown without salt had significantly less Na^+^ in the leaves than Wt plants, whereas the Na^+^ content of the flower stalks was the same (Figures 2A,C). However, the 14-3-3 mutant plants grown in low-Na^+^ soil had similar levels of Na^+^ in the leaves and flower stalks compared to Wt plants in the winter experiment (Figures 2B,D). Additionally, salt-treated Wt and 14-3-3 mutant plants showed a 2- to 3-fold increase in Na^+^ in both experiments. However, under both summer and winter conditions, the *klpc* mutant contained significantly less Na^+^ in the leaves and stalks as compared to Wt plants (35–45% less) (two-way ANOVA interaction, *P* < 0.001). As expected, the *hkt1* mutant showed strong reductions in K^+^ concentrations in the rosette leaves as compared to those in Wt plants, both in control and salt-stressed plants (*P* < 0.001, Figures 2E,F). However, in the flower stalks, there was little to no reduction in K^+^ accumulation (Figures 2G,H); this is noteworthy in view of the high level of Na^+^ accumulation in the *hkt1* flower stalks under all conditions (Figures 2A–D). How this differential flow of Na^+^ was achieved remains an intriguing question. In the 14-3-3 mutant plants, *klpc* showed a significant reduction in the K^+^ content of rosette leaves as compared to that in Wt leaves, both in control and salt-stressed conditions (Figures 2E,F). However, the K^+^ content of the *klpc* flower stalks was the same as those of Wt plants (Figures 2G,H).

#### Na^+^/K^+^ Ratio

**Figure 3:**
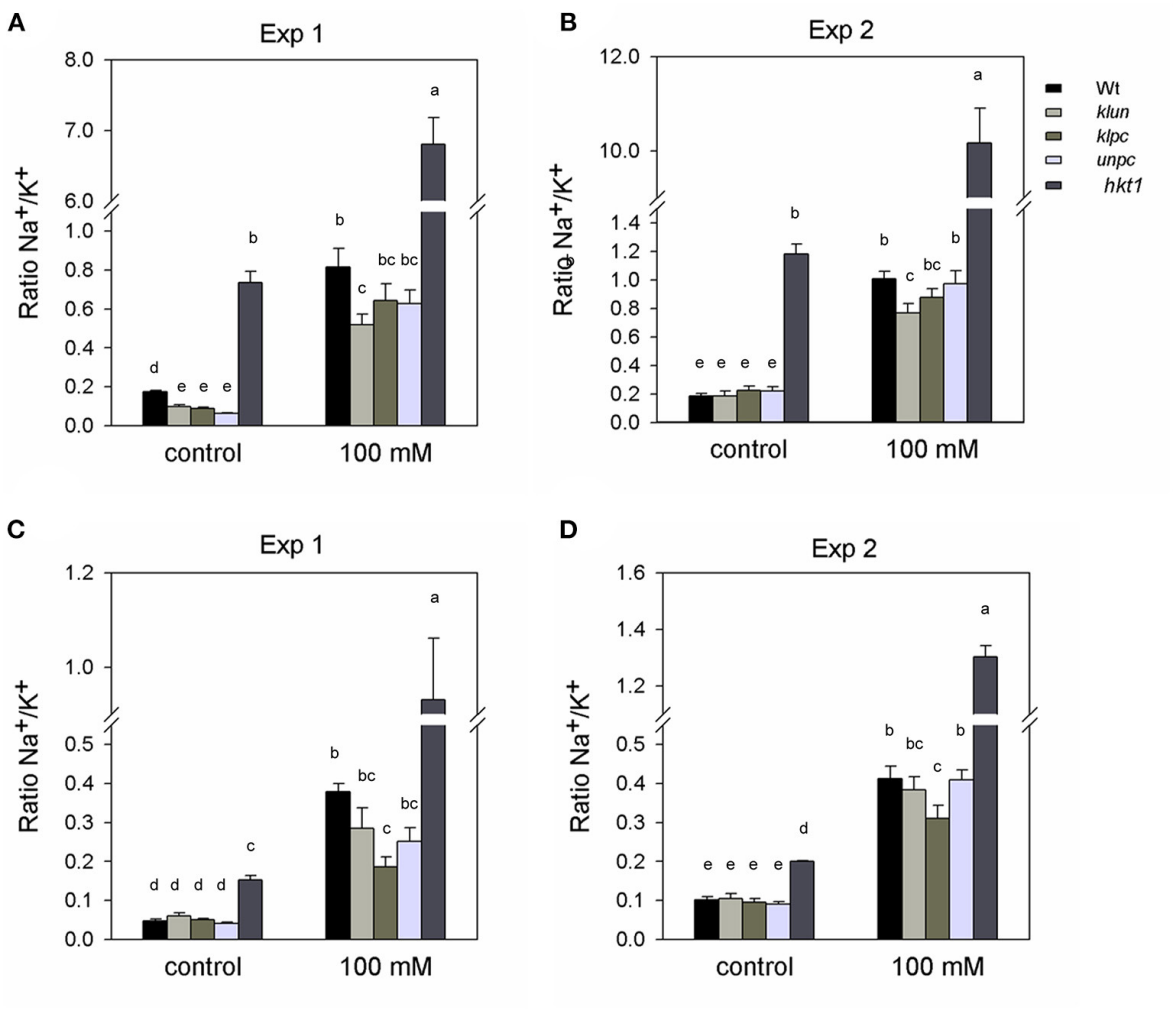
Na^+^:K^+^ ratio calculated for the rosette leaves and inflorescence of Wt, 14-3-3 qKOs, and *hkt1* mutant plants, grown with and without (= control) the addition of 100 mM of NaCl. Two independent experiments: Exp. 1 (in the winter) and Exp. 2 (in the summer). **(A,B)** Na^+^: K^+^ ratio of rosette leaves. **(C,D)** Na^+^: K^+^ ratio of inflorescence. Values are averages ± SE (*n* = 3~5). Different letters above the column indicate significant differences between the treatments or genotypes according to a two-way ANOVA (*P* < 0.05; Tukey's test). Statistics can be found in Supplementary Table 3.

Maintaining a low Na^+^/K^+^ ratio is a good strategy for plants to adapt to salt stress (Serrano and Rodriguez-Navarro, 2001). Figure 3 shows the Na^+^/K^+^ ratios as calculated from the data shown in Figure 2. The Na^+^/K^+^ ratio of *hkt1* plants was very high (ranging from 5 to 7), although this was not reflected in the salt-dependent reduction of growth (Figure 1). Under salt stress, leaves (not the stalks) of the *klun* plants showed a significantly lower Na^+^/K^+^ ratio than Wt plants (Figures 3A,B), whereas the flower stalks (not the leaves) of the *klpc* plants showed a significantly lower ratio than Wt plants (Figures 3C,D). Therefore, although we found clear differences in the Na^+^ and K^+^ accumulations and ratios between Wt and mutant plants, we did not find a correlation between ion homeostasis and growth performance under salt stress, not even for the *hkt1* mutant plants.

### Rate of Water Loss of 14-3-3 Quadruple Mutants

**Figure 4:**
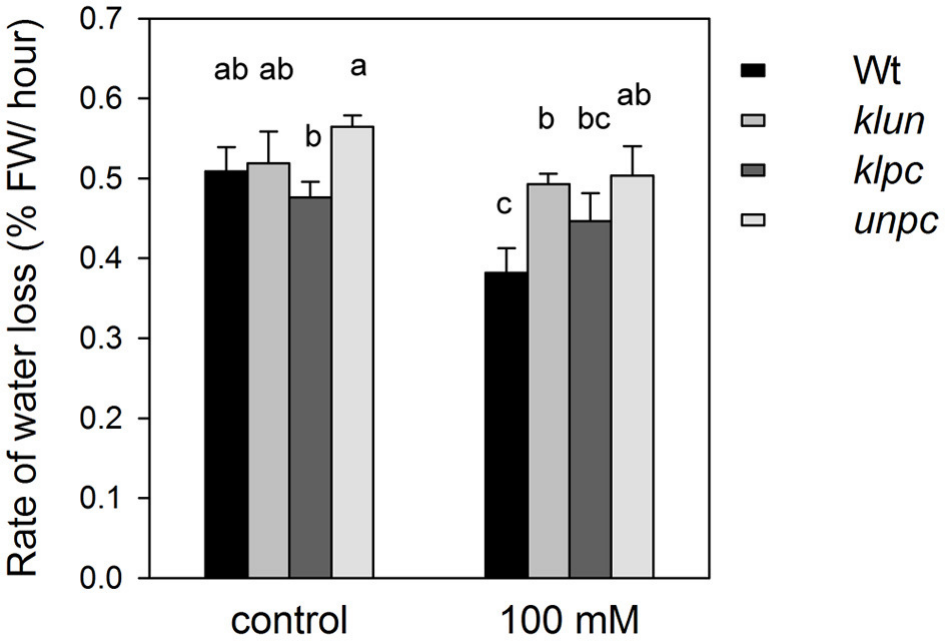
Water loss by transpiration of 24-day-old Wt and 14-3-3 qKO plants grown in soil and treated with 0 (= control) or 100 mM of NaCl for 3 h. Transpiration was calculated by monitoring the decrease in pot weight over several hours. Values are averages ± SE (*n* = 6). Different letters indicate significant differences between the treatments or genotypes according to a two-way ANOVA followed by Tukey's test. *F*_genotype_ = 6.936 (*P* = 0.015), *F*_treatment_ = 1.303 (*P* = 0.059), and *F*_genotype×*treatment*_ = 0.087 (*P* = 0.771).

Since the role of 14-3-3 proteins in stomatal opening and development is well-documented (Kinoshita et al., 2003; Tseng et al., 2012; Sun et al., 2014), the delivery of Na^+^ and K^+^ from roots to shoots by the transpiration stream might be affected by mutations in the 14-3-3 genes, e.g., the lower Na^+^ concentrations in the shoot of *klpc* plants could be the result of reduced stomatal openings and a lower overall transpiration rate. To test this hypothesis, Wt, *klun, klpc*, and *unpc* plants grown in the green house were exposed to 0 or 100 mM of NaCl for 3 h, and the rates of water loss (decrease in fresh weight per hour) were measured after 1 h. As shown in Figure 4, salt stress caused a significant decrease in the transpiration of the salt-treated Wt plants, while all three 14-3-3 qKO mutants maintained their transpiration rates despite the salt treatment. Therefore, transpiration differences between Wt and qKOs do not provide an explanation for the reduction in the Na^+^ accumulation in the *klpc* mutants.

### *AtHKT1* Expression Is Altered in the *klpc* Mutant

**Figure 5:**
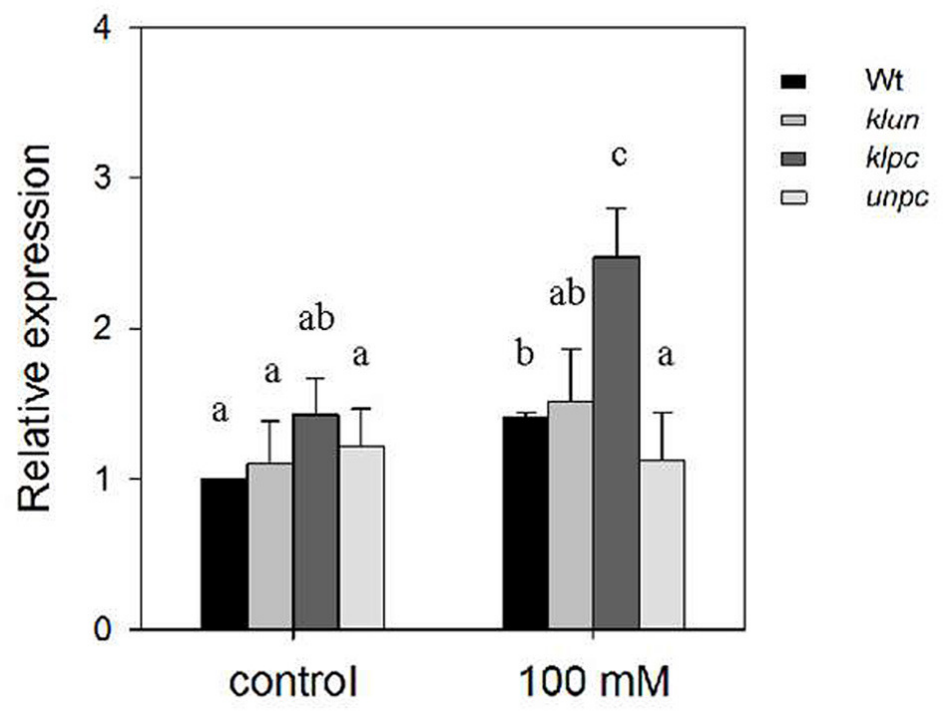
Relative expression of *HKT1* in the primary root of Wt and 14-3-3 qKO plants grown with and without 100 mM of NaCl. Soil-grown plants (aged 24 days) were treated with 0 or 100 mM NaCl for 1 day and then had their primary roots harvested (first 2 cm below the rosette leaves) and total RNA extracted. Each sample was assayed three times. Expression levels were normalized to Actin and then to the *HKT1* expression level of the Wt plants under control conditions. Different letters indicate the means ± SD, which are significantly different at *p* < 0.05 (ANOVA followed by Tukey's test, *n* = 5 biological replicates). *F*_genotype_ = 4.302 (*P* = 0.011), *F*_treatment_ = 42.132 (*P* < 0.001), and *F*_genotype×*treatment*_ = 1.522 (*P* = 0.234).

The analysis of the *hkt1* mutant plants (Figure 2) clearly showed the dramatic effect of the absence of a functional HKT1 protein on Na^+^ and K^+^ accumulations in the shoots. Therefore, if 14-3-3 proteins act as repressors of the salt-induced expression of *HKT1*, e.g., through their interaction with the SOS2 kinase (Zhou et al., 2014), and assuming a coordinating role for the activated SOS2 in *HKT1* expression (Wang et al., 2014b), then this might explain the observed changes in Na^+^/K^+^ homeostasis in the 14-3-3 mutants. Obvious physiological changes of plants require exposure to long-term high salt stress, but gene expression will respond within 24 h (Huang et al., 2012). Therefore, we measured the gene expression of *HKT1* in the primary roots of the Wt plants and 14-3-3 qKOs after 24-h exposure to high salinity by quantitative real-time PCR (qPCR). As shown in Figure 5, the *HKT1* transcript levels in Wt were significantly higher in Wt plants after treatment with NaCl (1.41-fold). The *HKT1* transcript levels in *unpc* and *klun* were also comparable to those in Wt plants, with and without salt stress. However, salt-treated *klpc* plants showed a more than 2-fold higher expression of *HKT1* compared to Wt plants (Figure 5). To avoid biased normalization under salt stress, UBQ5 was also used as a reference gene according to the recommendations of various abiotic stress studies. After salt treatment, the *HKT1* transcript levels in *klpc* were also higher than those in Wt and the other two 14-3-3 KOs, albeit not significantly (*p* > 0.05; Supplementary Figure 4). Therefore, we concluded that the reduction in the accumulation of Na^+^ in the shoots of *klpc* correlates with a higher level of *HKT1* expression in the main roots of this mutant. Furthermore, we found that the combination of the KLPC 14-3-3 proteins negatively regulates *HKT1* expression, either directly or indirectly.

### Metabolome Profiling of Wt and 14-3-3 qKOs Roots

Although salt stress is known to alter the levels of many metabolites (Hill et al., 2013) and 14-3-3 proteins play a crucial role in many metabolic pathways (Diaz et al., 2011; Swatek et al., 2011), little is known about the role of 14-3-3 proteins in salinity-induced changes of the metabolome. Since roots are the primary organs involved in ion homeostasis and salt tolerance for plants, we hypothesized that 14-3-3 proteins are instrumental in the salt-induced changes of the metabolite profile. Thus, we tested this assumption by generating the metabolic profiles of roots from Wt plants and the 14-3-3 qKOs. Because it was impossible to harvest all the roots of each plant from the soil, we switched to hydroponics (with a one-half strength Hoagland) to grow plants. After being exposed to 0 or 100 mM of NaCl for 24 h, the total root systems of the 14-3-3 qKO and Wt plants were harvested for an analysis of the metabolomics changes under salt stress. A total of 153 metabolites in the control and salt-treated plants were monitored by the GC-TOF-MS profiling of a metabolite fraction enriched for primary metabolites. This set included 80 known annotated metabolites and 73 known yet non-identified metabolites (Supplementary Table 1).

**Figure 6:**
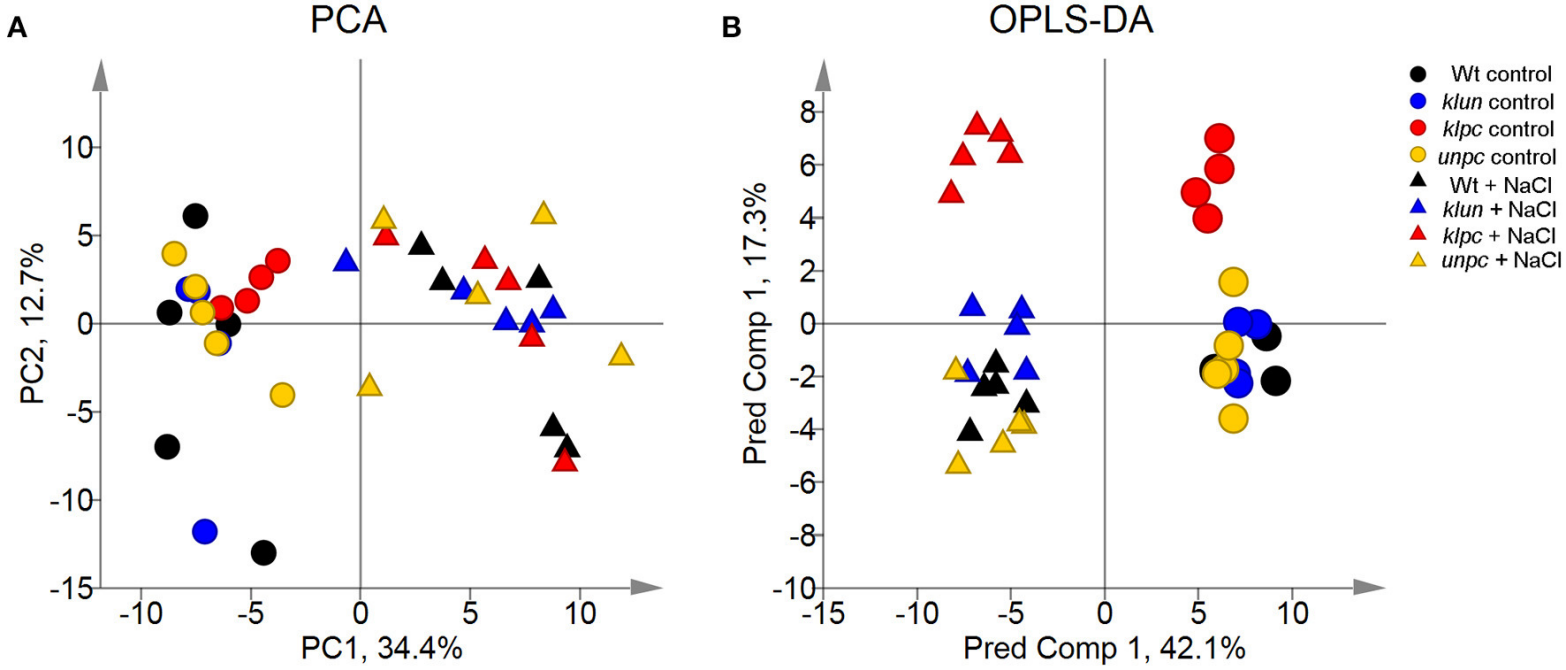
Principal component analysis (PCA) and orthogonal projections to latent structures discriminant analysis (OPLS-DA) score scatter plots of the metabolite profiles of *klpc, klun, unpc*, and Wt plants. **(A)** Principal component (PC) 1 and PC 2 represent the first two principal components accounting for a total of 47.1% of the variances. **(B)** The OPLS-DA model of the metabolic profiles shows three significant components with R2X, R2Y, and Q2 values of 0.639, 0.403, and 0.298, respectively. Log_2_-transformed response ratios based on the replicate medians of all measured metabolites relative to the Wt control were analyzed. Each condition was analyzed by 4~5 biological replicates. Missing values were replaced by 0 after log_2_-transformation. Different colored symbols indicate the different genotypes. Circle symbols, control samples; triangle symbols, salt-treated samples.

**Figure 7:**
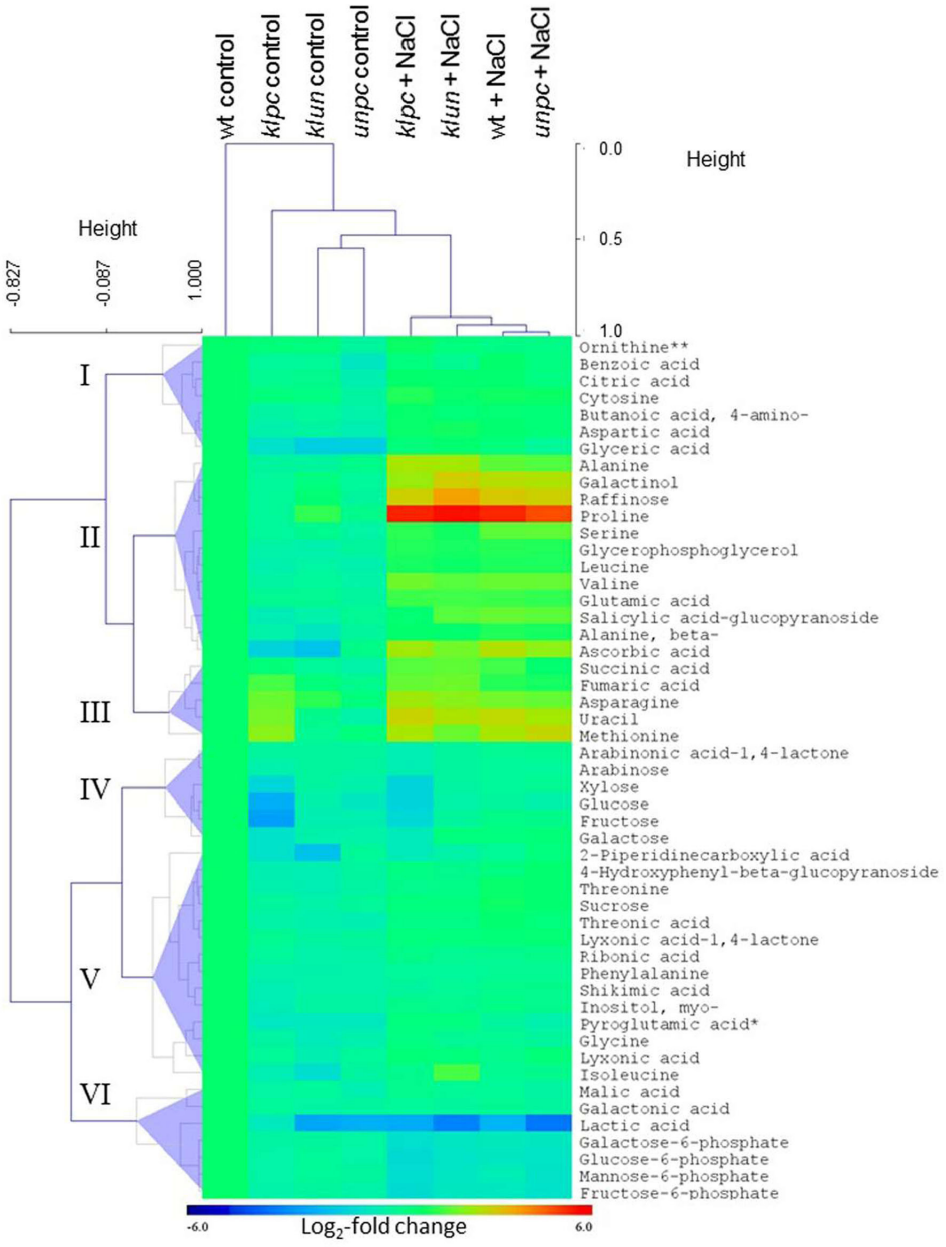
Pearson's correlation of log_2_-transformed response ratios comparing the metabolic salt stress responses of roots from the 14-3-3 quadruple mutants to the Wt salt stress response. Response ratios of *klun, klpc, unpc*, and Wt metabolite pools under salt stress were calculated relative to the Wt control and averaged across replicate measurements before log-transformation (*n* = 4–5). Each item represents a metabolite. The correlation coefficient (*r*^2^) of the Pearson's correlation and the linear equation are reported by inserts. Note the lower correlation coefficient (*r*^2^) and deviation of the linear slope from 1 of the comparison between *klpc* and Wt.

To visualize the metabolomic changes of Wt and the three 14-3-3 qKOs in response to salt stress, PCA and OPLS-DA of the complete metabolite profiling data were used to predict the way the samples were grouped and distributed (Figure 6). The non-supervised PCA scatter plot showed that the samples were clustered with respect to their treatments. The first two principal components (PC1 and PC2) accounted for a total of 47.1% of the variances. Subsequently, the supervised OPLS-DA revealed the clear separation between the *klpc* mutant from the Wt plants and the other mutants under both stressed and control conditions (Figure 6B). A combination of a two-way ANOVA, Mack–Skillings analyses, and the statistical testing of pairwise comparisons with all tests set to a threshold of *P* < 0.01 identified 51 known metabolites and 48 known yet non-identified compounds that were either stress responsive or relevant for the distinction among mutants and Wt. At the same significance threshold, we did not find apparent interactions between the factors of stress and genotype (Supplementary Table 1). We selected the subset of 51 relevant and known metabolites for an HCA applying Pearson's correlation and complete linkage to log_2_-transformed response ratios calculated relative to the mean Wt control (Figure 7). In agreement with the PCA analysis, HCA demonstrated that salt stress was the main factor of the experiment. Furthermore, the HCA of the relevant known metabolites confirmed that the central metabolism of the *klpc* mutant deviated most from Wt, *klun*, and *unpc* under both stressed and non-stressed conditions. We identified the six clusters (I–VI) of metabolites that represent the different response patterns of metabolites to salt stress across genotypes (Figure 7). Clusters I–III contained metabolites that accumulated in response to salt stress, whereas clusters IV–VI contained metabolites that did not change or decreased relative to control conditions. Notably, several metabolites, such as proline, galactinol, raffinose, and ascorbic acid, of cluster II accumulated highly across all tested genotypes. However, less extremely accumulating metabolites sorted into cluster I. Furthermore, the phosphorylated sugars of cluster VI, e.g., glucose-6P and fructose-6P, commonly depleted in response to salt stress. Metabolites that decreased less in response to salt stress also partitioned into clusters IV and V. Additionally, several clusters indicated mutant-specific metabolic changes under control conditions and specifically highlighted the changes caused by the *klpc* mutation. Cluster III also contained metabolites that accumulated in *klpc* under control conditions and remained upregulated under salt stress. Inversely, cluster IV contained metabolites that decreased in *klpc* under control conditions and remained decreased under salt stress. In summary, this analysis confirmed the largely common metabolic changes in salt-stressed roots across the genotypes but indicated characteristics and, in the case of *klpc*, performed metabolic changes under control conditions.

### 14-3-3 qKO Specific Metabolic Changes

**Figure 8:**
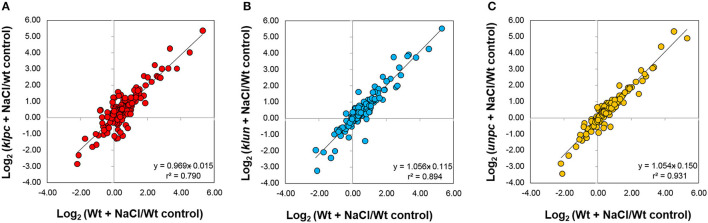
Hierarchical cluster analysis (HCA) and the heat map of metabolite profiles of the *klpc, klun*, and *unpc mutants* and Wt plants. HCA was performed using log_2_-transformed response ratios relative to Wt control means (*n* = 4–5). Pearson's correlation (*r*) and complete linkage were applied. The analysis was based on 51 known metabolites selected at *P* < 0.01 of either a two-way ANOVA, Mack-Skillings tests, or heteroscedastic Student's *t*-tests (Supplementary Table 1). Clusters I–VI were defined below half the height of the metabolite clustering tree. The color key indicates the relative changes in metabolites compared to Wt plants (Log_2_-fold changes). Note the similarity of metabolite profiles from *klun, unpc*, and Wt plants under salt stress and control conditions and the corresponding dissimilarity of *klpc* plants. **Due to the constraints of the GC-MS-based metabolite profiling, ornithine represents the sum of arginine, citrulline, and ornithine.

These observations prompted us to assess the degree of commonality between Wt responses to salt stress and the respective 14-3-3 qKO mutant responses. For this purpose, we analyzed the bi-plots of the salt-induced log_2_-transformed relative changes in the mutants compared to Wt (Figure 8). This analysis revealed that the *klpc* mutant had the most divergent salt stress response, according to Pearson's correlation, *r*^2^ = 0.79, in comparison to Wt plants. In contrast, the *klun* and *unpc* salt responses were more similar to the Wt, as was indicated by *r*^2^ = 0.894 and 0.931, respectively (Figure 8).

To visualize the induced production of metabolites in response to salinity, we selected metabolites that were significantly changed (*P* < 0.01, heteroscedastic Student's *t*-test) compared to Wt plants cultivated under control conditions (Table 1). This analysis revealed that 30 known metabolites changed significantly in at least one genotype in response to salt treatment. By far, most of the salt-responsive metabolites accumulated and were common among Wt and the mutants, e.g., the top-ranking proline, galactinol, raffinose, and ascorbic acid (Figure 7 and Table 1), while only a few metabolites decreased according to this selection, e.g., mannose-6P and galactose-6P. These metabolites were already reduced by tendency in Wt, but became significantly reduced in the 14-3-3 qKO mutants (*P* < 0.01) compared to the Wt control. Furthermore, the general metabolic shifts of 14-3-3 qKOs and Wt were highly similar, but several attenuations or losses of significance were detected in the 14-3-3 qKOs, e.g., galactonic acid and shikimic acid in all three qKOs, alanine in *klun* and *unpc*, and salicylic acid-glucopyranoside that accumulated less in *klpc*.

**Table 1:**
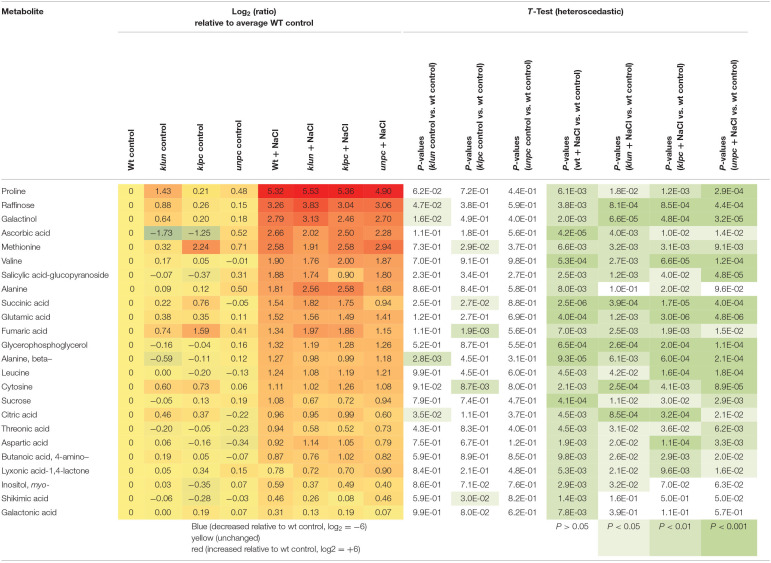
Salt-responsive metabolites of Wt roots compared to *klun, klpc*, and *unpc* mutant roots.

*Salt-responsive metabolites were selected at P <0.01 (heteroscedastic T-Test) observed either in mutants or Wt and were sorted in descending order according to the magnitude and direction of the change in salt-stressed Wt roots. The green color means significance levels by heteroscedastic T-Test*.

To identify the specific differences among the metabolic responses to NaCl, the metabolite profiles of Wt plants and 14-3-3 qKOs were evaluated for differential salt-responsive metabolites (Supplementary Table 1). At this significance threshold, we found no common difference of all mutants vs. Wt. We only found a single significant change each for *klun* and *unpc*, namely, sucrose and succinic acid, respectively. In contrast, nine known metabolites (Table 2) and three non-identified compounds (Supplementary Table 1) were different for *klpc*. Fructose, glucose, and several minor carbohydrates, such as galactose, arabinose, and xylose, were, in part, more than 2-fold decreased under salt stress relative to the Wt control. Many of these changes were already present and more extreme under control conditions, e.g., fructose, glucose and galactose (Table 2). Furthermore, a subsequent analysis of mutant-specific metabolic changes under control conditions revealed five differential known metabolites (Table 3) and seven known yet non-identified compounds (Supplementary Table 1). Most of the preformed metabolic changes were observed in *klpc*, namely, asparagine, fumaric acid, cytosine, and fructose, as expected (Table 2). However, *unpc* had no significant difference to Wt under control conditions. *klun*, on the other hand, had decreased beta-alanine levels, shared accumulation of asparagine, and a tendency for fumaric acid and cytosine under control conditions (Table 3).

**Table 2:**
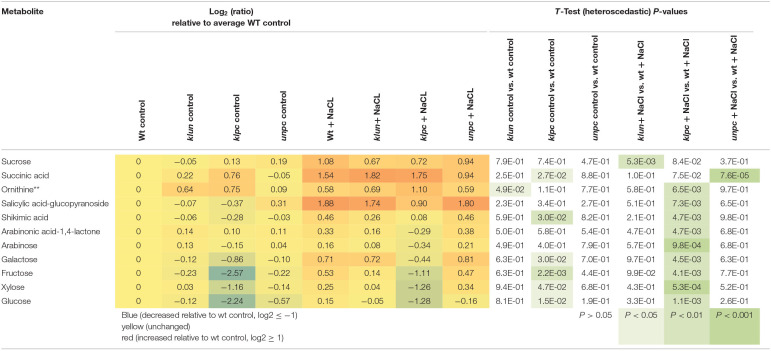
Differential metabolic response to salt stress in *klun, klpc*, and *unpc* mutant roots compared to Wt. Log_2_ (ratios) and significance values (*P* < 0.01, heteroscedastic *T*-Test) are listed.

*The green color means significance levels by heteroscedastic T-Test*.

**Table 3:**
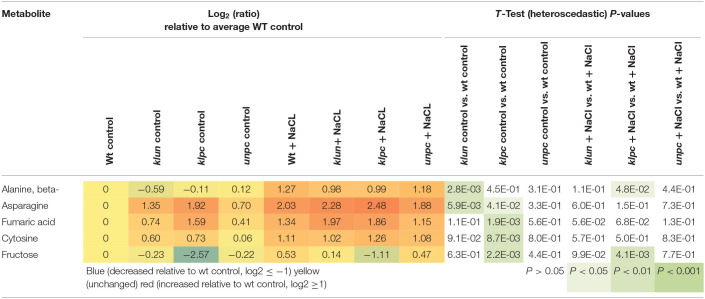
Differential metabolic changes in *klun, klpc*, and *unpc* mutant roots compared to Wt under control conditions.

*Log_2_ (ratios) and significance values (P <0.01, heteroscedastic T-Test) are listed. The green color means significance levels by heteroscedastic T-Test*.

Taken together, the supervised statistical analyses confirmed and refined the global observations made by the PCA and HCA analyses. Our analyses suggested that the metabolic responses to salinity between the Wt and 14-3-3 qKOs were largely similar, and that preformed isoform-specific differences exist at the metabolome level in the *klpc* mutant.

### Exemplary Differential Metabolites of *klpc*

**Figure 9:**
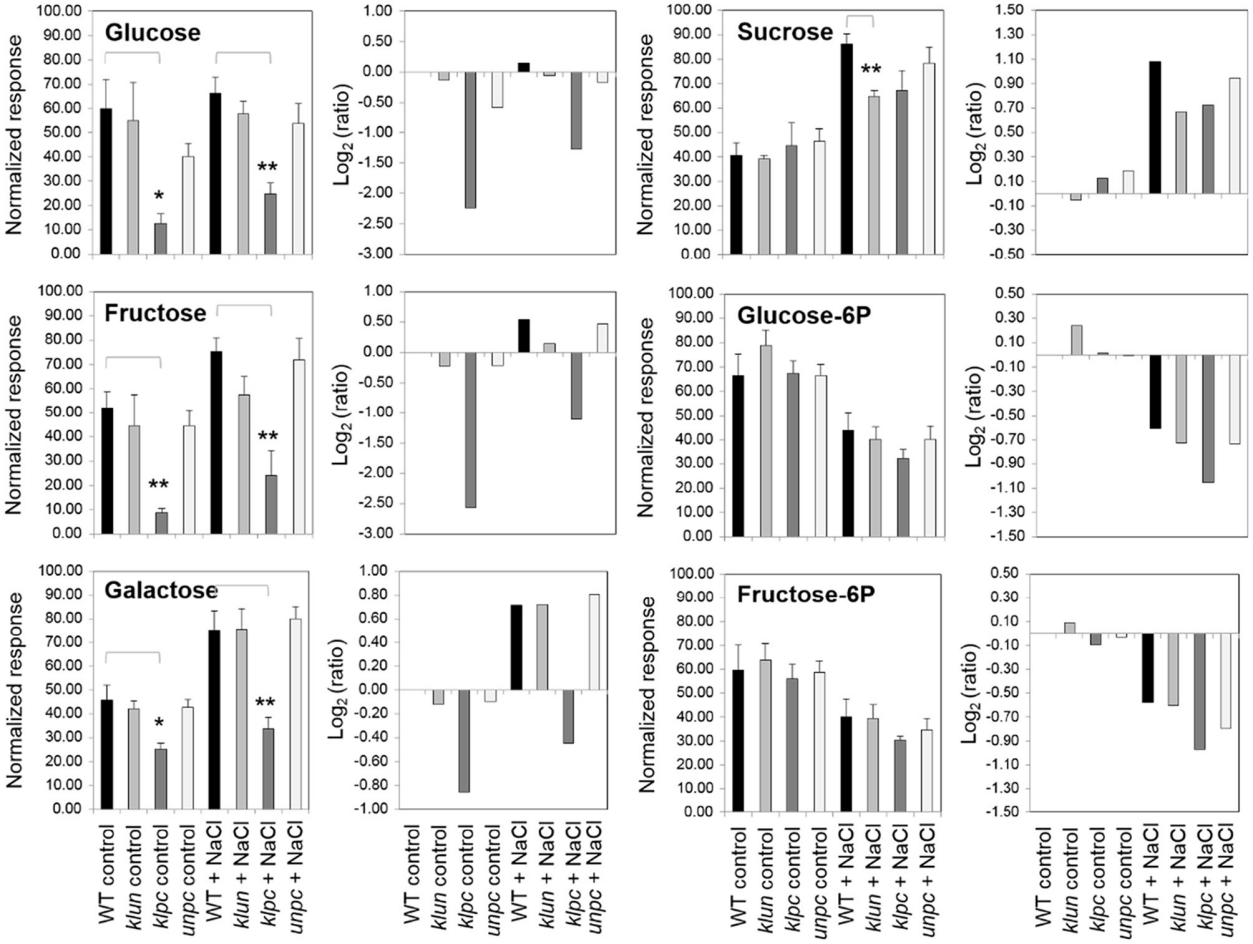
Changes in selected metabolites from *klun, klpc*, and *unpc* mutant roots compared to Wt under control and salt stress conditions. Normalized responses (left panels, mean ± standard error) and log_2_-transformed response ratios (right panels) were calculated relative to the Wt control. Bar diagrams show medians and standard errors. Stars indicate significant changes compared to the Wt as indicated by brackets (*P < 0.05, **P < 0.01, heteroscedastic *T*-Test). Note the specific constitutive changes of glucose, galactose, and fructose levels in *klpc* plants compared to the largely common salt-induced responses of sucrose, glucose-6P, and fructose-6P.

The metabolic defect of the *klpc* mutant pointed to a dysregulation of the carbohydrate metabolism, however, with no direct consequences of 14-3-3 qKO mutations for the accumulation of sucrose and decreases of glucose-6P and fructose-6P in roots under salt stress (Figure 9). In contrast to these salt-regulated metabolites, the non-salt-responsive sugars, glucose and fructose, were specifically reduced in *klpc* mutant roots. This more than 2-fold decrease of glucose and fructose was highly specific to *klpc* (Figure 9) and extended only in part to minor carbohydrates. The lack of glucose and galactose was associated with the reduced accumulation of salicylic acid-glucopyranoside (Table 2) but not by altered galactinol or raffinose accumulations under salt stress.

## Discussion

Proteins from the 14-3-3 family are important regulators of ion homeostasis and the primary nitrogen/carbon metabolism. They have also been reported to play a role in abiotic stress adaptation (Huber et al., 2002; Diaz et al., 2011; De Boer et al., 2013). In this study, we used different 14-3-3 mutants to demonstrate the relevance of 14-3-3 proteins in the salt stress adaptation of *Arabidopsis*. Since there was a likely redundancy between the members of the 14-3-3 family, we selected six members from the non-epsilon group of the *Arabidopsis* 14-3-3 family and generated three quadruple knockout mutants by crossing double mutants made of closely related gene pairs (Van Kleeff et al., 2014). First, we phenotyped these mutants for salinity-affected growth and Na^+^/K^+^ ion homeostasis when grown in soil under natural greenhouse conditions. Next, we analyzed the root metabolome of the mutants grown in one-half strength Hoagland as affected by salt stress and compared this with the Wt metabolome.

To validate our method of salt stress application, we included the Na^+^-hyper-accumulating mutant *hkt1*. As shown in Figure 2, *hkt1* accumulated large amounts of Na^+^ not only in salt treated pots, but even from the control pots. This “sponge-like” behavior with respect to shoot Na^+^ accumulation was even observed when *hkt1* mutants were grown on a Hoagland solution with minimal amounts of Na^+^ (Hill et al., 2013). Surprisingly, despite the much higher accumulation of Na^+^ and stronger reduction in K^+^ levels in the rosette leaves, the *hkt1* mutant plants performed similar to the Wt plants. In another study, the growth of soil-grown *hkt1* mutant plants was strongly reduced by salt; however, in this study, the salt exposure lasted 6 weeks (Rus et al., 2006). Moreover, the strong reduction in rosette leaf K^+^ of the *hkt1* mutant was not reflected in the K^+^-levels of the flower stalks. How this mutant allocated enough K^+^ to the flower stalk when the leaf K^+^ was much lower remains an open question. The extremely high Na^+^/K^+^ ratios in the *hkt1* mutant, combined with a lack of growth inhibition, warrant further studies.

The biomass productions of all genotypes were equally reduced by the salt treatment, specifically, a reduction of around 30–40%. *unpc* seemed to be more affected by salt than Wt. The *klpc* mutant also stood out when looking at Na^+^ and K^+^ homeostasis under salt stress, as it contained significantly less Na^+^ in the rosette leaves and flower stalks as compared to Wt. K^+^ levels in the *klpc* leaves were also lower compared to Wt leaves, but the flower stalks maintained K^+^ levels equal to those in Wt stalks (Figure 2). Therefore, the *klpc* mutant showed a clear Na^+^ and K^+^ phenotype. Furthermore, van Kleef et al. have shown that double mutant *un* showed fewer reductions in primary root growth in a 100-mM NaCl treatment, while *kl* and *pc* were indistinguishable from Wt (Van Kleeff et al., 2014). Whether the different root morphology changes can also be observed in the three quadruple mutants needs further study. In the present study, however, neither the combination of *kl* with *un* (*klun*), nor *un* with *pc* (*unpc*) showed the same phenotype as the *klpc* mutant, suggesting that there was specificity among the 14-3-3 genes tested. Whether there was redundancy between the four 14-3-3 *KLPC* genes can also be tested by analyzing the triple, double, and single mutants.

The lower levels of Na^+^ and K^+^ in *klpc* were not due to a reduced transpiration rate under salt stress, but correlated with an increase in the expression of the *HKT1* gene in this mutant (Figure 5). A model wherein a specific set of 14-3-3 proteins acts as repressor of *HKT1* expression could explain this observation. Additionally, HKT proteins counteract the activation of the Salt Overly Sensitive (SOS) pathway for salt tolerance (Katschnig et al., 2015). Na^+^ and K^+^ homeostasis in *Arabidopsis* under salt stress has also been found to be improved by the coordinated expression of *AtHKT1;1* and *AtSOS1* (Wang et al., 2014b). Furthermore, 14-3-3 acts as an upstream regulator of the SOS pathway *via* not only interacting with and inhibiting SOS2 activity, but also reducing SOS1 transporter activity (Duscha et al., 2020). It has also been reported that members of the non-epsilon group, 14-3-3 KAPPA and LAMBDA, interact with and repress the activity of SOS2 in the absence of salt (Zhou et al., 2014; Yang et al., 2019). When plants were exposed to salt, the SOS2/14-3-3 interaction was reduced and SOS2 kinase activity enhanced. It may be more than a coincidence that KAPPA and LAMBDA are also represented in the *klpc* 14-3-3 qKO that shows this reduced Na^+^ accumulation phenotype.

We used multivariate data analyses (PCA, OPLS-DA, and HCA models) to extract interpretable information from the multidimensional data generated by the GC-TOF-MS analysis. The global view as depicted by the PCA and OPLS-DA scores managed to separate control and salt-treated samples into distinct groups (Figure 6). The HCA also gave a separation quite similar to the one seen in the OPLS-DA, as shown in Figure 7, as control and salt-treated samples were again found in distinct groups. Furthermore, the HCA showed that salt stress was the main effector on root metabolism. Also, the heat map suggested that the salt stress response of the mutants was, in general, similar compared to Wt, while several metabolites showed *priori* differences compared to Wt (*klpc* in cluster III and V).

The mutant that differed most from the salt stress responses of Wt in terms of metabolite composition was the *klpc* mutant (Figure 8). The most notable differences were the lower levels of fructose, glucose, and galactose (Figure 9). The reduction in glucose and fructose levels could be assigned to the positive regulation of root alkaline invertase by 14-3-3 proteins (Gao et al., 2014). A number of other enzymes with a function in carbohydrate metabolism have also been reported to be controlled by 14-3-3 proteins, such as sucrose phosphate synthase (SPS), 6-phosphofructo-2-kinase/fructose-2,6-bisphosphatase (6PF2K), and trehalose-phosphate synthase (TPS) (Kulma et al., 2004; Bornke, 2005; Harthill et al., 2006). This may explain why, in a previous 14-3-3 over-expression study, 14-3-3KAPPA-OX and 14-3-3CHI-OX plants also showed a reduction in sugars (sucrose, glucose, and fructose), although these measurements were done on shoot material (Diaz et al., 2011).

Differences in the mutant metabolomes vs. those of the Wt as seen under control conditions all disappeared (except for the sugars and some acids in *klpc*) when the plants were salt stressed, e.g., cytosine, fumaric acid, and raffinose (Supplementary Figure 5). One explanation for this is that, under salt stress, the mutants compensated for the loss of some of the 14-3-3 proteins through the salt-induced upregulation of redundant 14-3-3 genes. Such an upregulation of 14-3-3 genes by salt was also observed in cotton (Wei et al., 2009) and tomato roots (Xu and Shi, 2006).

As expected, each genotype showed a multitude of salt-induced metabolic changes (Table 2). Global metabolic changes upon high salinity included increases in the levels of amino acids, sugars, and polyols, which have been previously identified as stress-responsive metabolites. In our experiments, the response of most metabolites was the same for Wt and 14-3-3 qKOs, but the mutants accumulated different amounts of salts (Figure 2). We also saw an increase in the levels of many amino acids and sugars, while the *klpc* mutant showed significant reductions in intermediates of glycolysis (Table 1 and Figure 9). The latter fit the conclusion that we reached in a review that 14-3-3 is a key regulator of glycolysis through interactions with many individual glycolytic enzymes and/or enzyme complexes (De Boer et al., 2013). Thus, glycolytic regulation is evidently complex, as the over-expression of single 14-3-3 genes (*KAPPA* and *CHI*) induced a strong reduction in the level of key metabolites in glycolysis (Diaz et al., 2011). The physiological role of the salt-responsive metabolites that are specifically changed in *klpc* remains to be investigated. Furthermore, the observation from Figure 9 indicated that more sucrose accumulated in roots under salt stress while the levels of glucose-6P and fructose-6P declined. Raffinose biosynthesis requires sucrose and galactose that are conjugated to myo-inositol to form galactinol. The galactose moiety is then transferred as the galactinol intermediate to sucrose for raffinose production. In these aspects, the *klpc* mutant roots largely do not differ from salt-stressed Wt and the other mutants, likely meaning that it is able to compensate for its defect concerning the 14-3-3-driven activation of the cytosolic invertase AtCINV1 (Gao et al., 2014). Additionally, *Arabidopsis* compensated for this dysregulation, which only had a few pleiotropic effects, e.g., the accumulation of ornithine that, due to the constraints of GC-MS-based metabolite profiling, represents the sum of urea cycle metabolites, arginine, citrulline, and ornithine (Table 1). It may be that this compensation reflects the lower cellular stress in *klpc* (due to the reduced Na^+^-accumulation) compared to Wt. Alternatively, these metabolites may rather be involved in other physiological processes such as signaling or act as repositories of carbon and nitrogen. Whether this compensation invests resources into enhanced growth and less Na^+^ accumulation under salt stress needs to be further investigated. The over-expression of cytosolic invertase under a salt-inducible promoter may also prove important for future functional analyses. Clearly, the non-supervised and supervised analyses in this study showed differences in metabolite levels between *klpc* and Wt. The question on whether the differences in salt-induced changes in specific metabolite productions can be separated from the higher level of *HKT1* expression and reduced Na^+^-accumulation as observed in the *klpc* quadruple mutant may be answered by the analyses of the same parameters in the triple, double, and single mutants.

In conclusion, we addressed research questions regarding the role of higher order *Arabidopsis* 14-3-3 mutants in ion homeostasis and metabolite composition, both in control and salt-stressed conditions. The metabolomic analysis revealed that the 14-3-3 qKOs mutants had different metabolic profiles compared to Wt and that, notably, the metabolome of *klpc* plants under control and salt-stressed condition differed from that in Wt. These observations indicate that 14-3-3 proteins directly or indirectly affect the amino acid metabolism, TCA cycle, and carbohydrate metabolism both under non-stressed conditions and long-term salt stress. Our results confirmed that 14-3-3 proteins are involved in multiple salt stress-related signaling pathways, with both broad redundancy and specificity considering the tested non-epsilon 14-3-3 members. Further analyses of single, double, and triple mutants that constitute subsets of the most effective qKO mutant (*klpc*) may reveal details of the redundancy that appears to control ion homeostasis and the associated metabolic phenotypes.

## Data Availability Statement

The original contributions presented in the study are included in the article/Supplementary Material, further inquiries can be directed to the corresponding author/s.

## Author Contributions

AB conceived and designed the experiments. JG and MB performed the experiments. PK generated the 14-3-3 mutant plants. AE and JK performed the analysis of the metabolomics analyses. JG wrote the manuscript with suggestions by AB, JK, and DH. All authors read and approved the final manuscript.

## Funding

The project was supported by a grant from the Netherlands Organization for Scientific Research (NWO; 817.02.006) to AB and a grant from the National Natural Science Foundation of China (No. 31802146) to JG.

## Conflict of Interest

The authors declare that the research was conducted in the absence of any commercial or financial relationships that could be construed as a potential conflict of interest.

## Publisher's Note

All claims expressed in this article are solely those of the authors and do not necessarily represent those of their affiliated organizations, or those of the publisher, the editors and the reviewers. Any product that may be evaluated in this article, or claim that may be made by its manufacturer, is not guaranteed or endorsed by the publisher.

## Abbreviations

qKOs: quadruple knockout mutants
Exp: experiment
SOS: Salt Overly Sensitive
PCA: principal component analysis
OPLS-DA: orthogonal projections to latent structures discriminant analysis
HCA: hierarchical cluster analysis.
