# Supplementary material for: Ion Homeostasis and Metabolome Analysis of Arabidopsis 14-3-3 Quadruple Mutants to Salt Stress
Source: Front Plant Sci. 2021 Sep 13;12:697324. doi: 10.3389/fpls.2021.697324 (PMC8473882; doi:10.3389/fpls.2021.697324)
Supplement: Supplementary file 1 [file Data_Sheet_1.zip › Data Sheet 1 (101) Modified.docx]

Ion homeostasis and metabolome analysis of *Arabidopsis* 14-3-3 quadruple mutants to salt stress

J. Gao^1,2^, P.J.M. van Kleeff^2, 4^, M.H. de Boer^2^, A. Erban^3^, J. Kopka^3^, D. K. Hincha^3†^ and A.H. de Boer^2,5^*

^1^ Institute of Apicultural Research, Chinese Academy of Agricultural Sciences, 100093, Beijing, China

^2^ Department of Structural Biology, Faculty of Earth and Life Sciences, Vrije Universiteit Amsterdam, De Boelelaan 1085, 1081 HV, Amsterdam, the Netherlands;

^3^ Max-Planck-Institute Molecular Plant Physiology, Am Mühlenberg 1, D-14476, Potsdam, Germany;

^4^ Swammerdam Institute for Life Sciences, Department of Plant Physiology, University of Amsterdam, Kruislaan 318, 1098 SM, Amsterdam, the Netherlands

^5^ Department of Medicinal Chemistry, Beta Faculty, Vrije Universiteit Amsterdam, De Boelelaan 1108, 1081 HZ, Amsterdam, the Netherlands

† We dedicate this paper to the memory of Dirk K. Hincha

* Correspondence: ahdeboer54@gmail.com; Tel.: +31-20-5987162

*** Correspondence:**Albertus H. de Boer
ahdeboer54@gmail.com

**Supporting Information**

**Fig. S1. Characterization of 14-3-3 T-DNA insertion quadruple lines. A.** Overview of the quadruple mutants used and generated during this study. The inner hexagon depicts the single mutants in yellow and at the corners in grey are the double mutants. The outer triangle shows atthe corners in red are the quadruple mutants. **B.** Transcript PCRs of qKO transgenic plants. Amplification of genomic DNA confirmed the T-DNA insertion in *klpc*, *unpc* and *klun*. Total RNA was isolated and transcripts were detected using RT-PCR. **C.** Western blot showing 14-3-3 protein level in roots extract of 14-3-3 qKO transgenic plants. 2 μg roots extracts from each line were running on a 10% SDS-PAGE gel.

**Fig S2. Representative photographs of plants grown in greenhouse after treatment with 0 or 100 mM NaCl.** The photographs were taken after 1-week exposure to salt in experiments conducted in summer.

**Fig S3. Salt induced reduction of FW relative to FW of plants grown under control condition.** Two independent experiments were done: Exp.1 (in the winter) and Exp.2 (in the summer). Values are mean of fresh weight reduction induced by salt stress / mean biomass of the plants grown under control condition.

**Fig.** **S4.** **The relative expression of *HKT1* that referred to UBQ5 in the primary root of Wt and 14-3-3 qKO plants.** Soil grown plants (age 24 d) were treated with 0 or 100 mM NaCl for 1 day and then the primary root was harvested (first 2 cm below the rosette leaves) and total RNA was extracted. Each sample was assayed three times. Expression levels were normalized to UBQ5 and then to the HKT1 expression level of the Wt under control conditions. Different letters indicate the means ± sd (standard deviation) are significantly different at p < 0.05 (ANOVA followed by Tukey's test, n = 3 biological replicates)

**Fig. S5. Exemplary metabolites in *klun*, *klpc* and *unpc* mutant roots compared to Wt under salt stressed and non-stressed control conditions.**

**Table S1.** ***F* values and significance levels for two-way ANOVA of the data for fresh weight and length of flower stem of different genotypes plants (Wt, *klpc*, *klun*, *unpc*, *hkt1*) under Na (0 and 100 mM) treatment.**

**Table S2. *F* values and significance levels for two-way ANOVA of the data for Na content and K content in different genotypes plants (Wt, *klpc*, *klun*, *unpc*, *hkt1*) under Na (0 and 100 mM) treatment.**

**Table S3. *F* values and significance levels for two-way ANOVA of the data for Na^+^ : K^+^ ratio in different genotypes plants (Wt, *klpc*, *klun*, *unpc*, *hkt1*) under Na (0 and 100 mM) treatment.**

**Table S4. Metabolite profiling data and statistical assessments (in separated profile).**

**A B C**


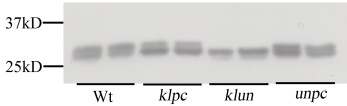

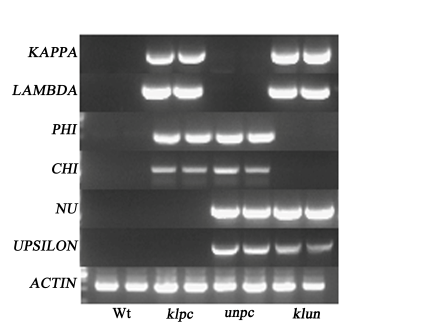

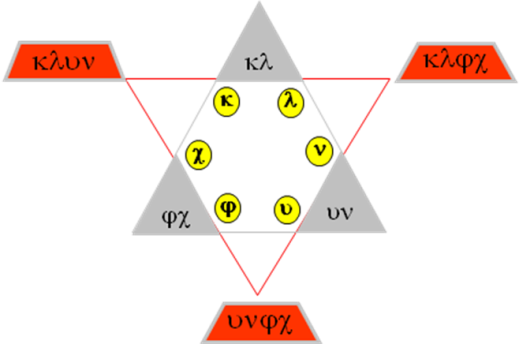


**Fig. S1. Characterization of 14-3-3 T-DNA insertion quadruple lines. A.** Overview of the quadruple mutants used and generated during this study. The inner hexagon depicts the single mutants in yellow and at the corners in grey are the double mutants. The outer triangle shows atthe corners in red are the quadruple mutants. **B.** Transcript PCRs of qKO transgenic plants. Amplification of genomic DNA confirmed the T-DNA insertion in *klpc*, *unpc* and *klun*. Total RNA was isolated and transcripts were detected using RT-PCR. **C.** Western blot showing 14-3-3 protein level in roots extract of 14-3-3 qKO transgenic plants. 2 μg roots extracts from each line were running on a 10% SDS-PAGE gel.


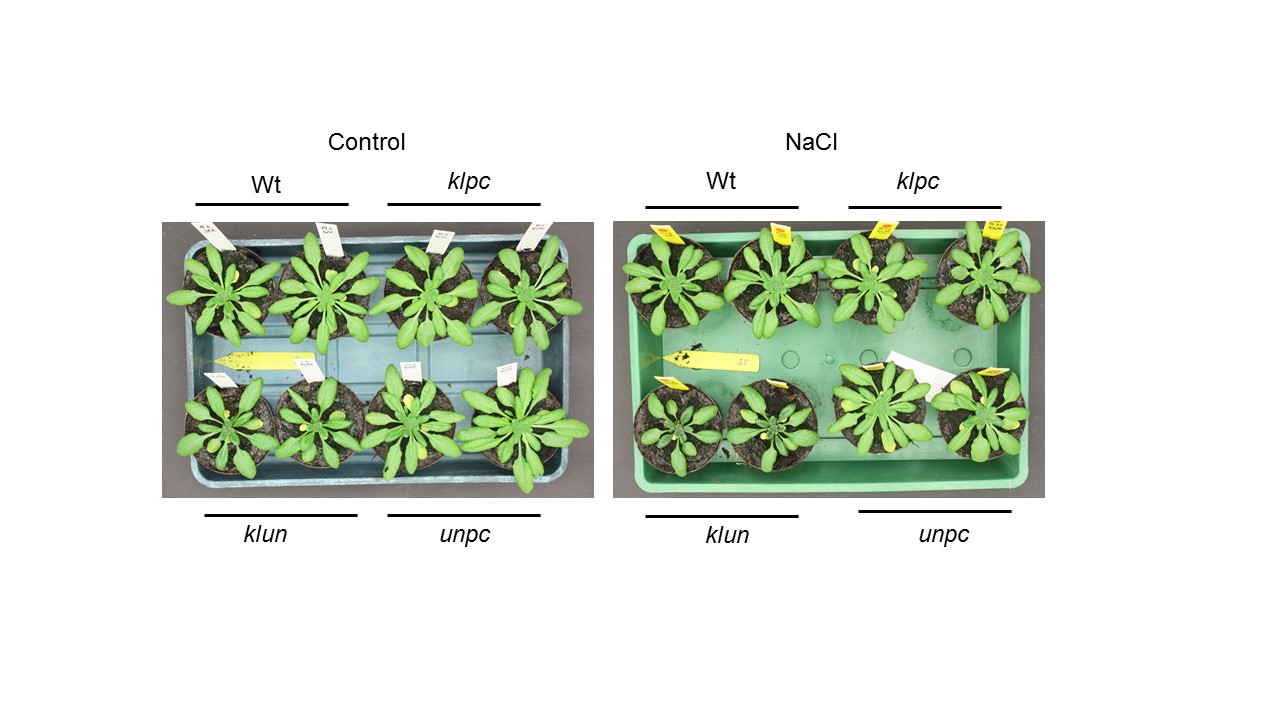


**Fig S2. Representative photographs of plants grown in greenhouse after treatment with 0 or 100 mM NaCl.** The photographs were taken after 1-week exposure to salt in experiments conducted in summer.


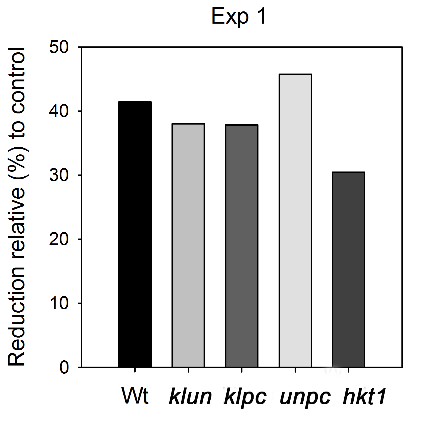

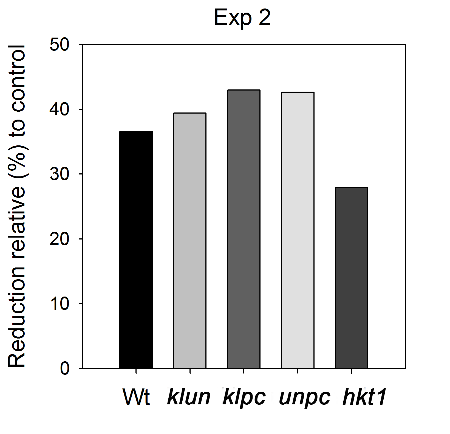


**Fig S3. Salt induced reduction of FW relative to FW of plants grown under control condition.** Two independent experiments were done: Exp.1 (in the winter) and Exp.2 (in the summer). Values are mean of fresh weight reduction induced by salt stress / mean biomass of the plants grown under control condition.

a

a

b

c

c

cd

c

c





**Fig. S4. The relative expression of *HKT1* that referred to UBQ5 in the primary root of Wt and 14-3-3 qKO plants.** Soil grown plants (age 24 d) were treated with 0 or 100 mM NaCl for 1 day and then the primary root was harvested (first 2 cm below the rosette leaves) and total RNA was extracted. Each sample was assayed three times. Expression levels were normalized to UBQ5 and then to the HKT1 expression level of the Wt under control conditions. Different letters indicate the means ± sd (standard deviation) are significantly different at p < 0.05 (ANOVA followed by Tukey's test, n = 3 biological replicates)


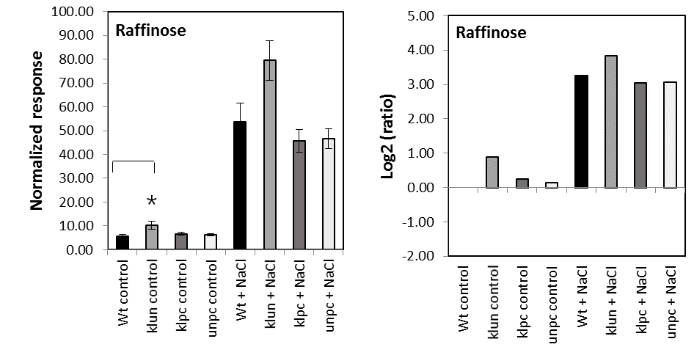


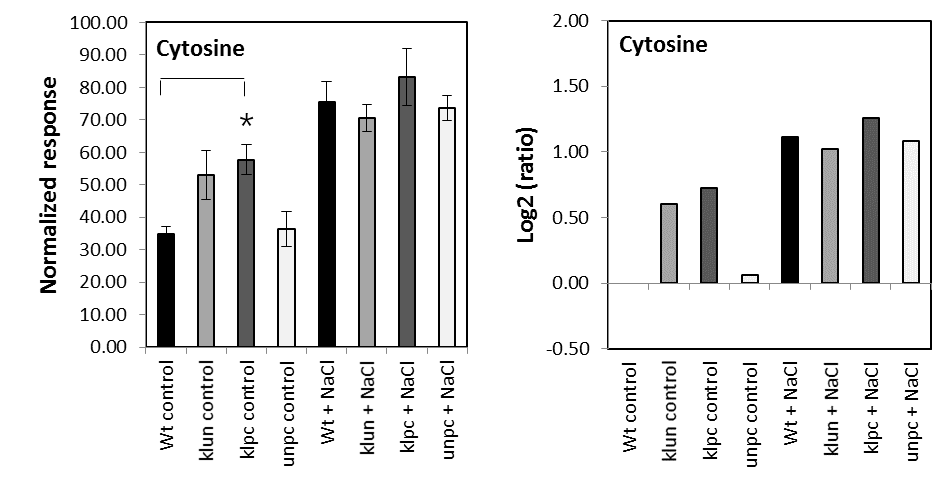

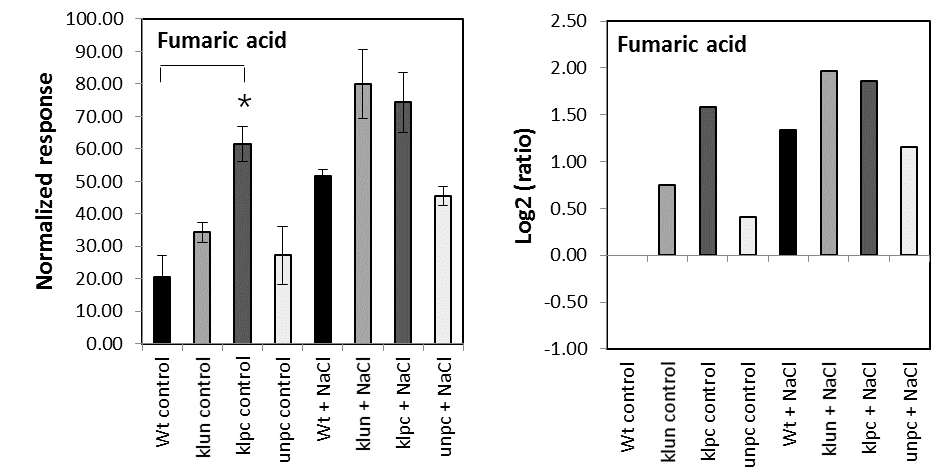


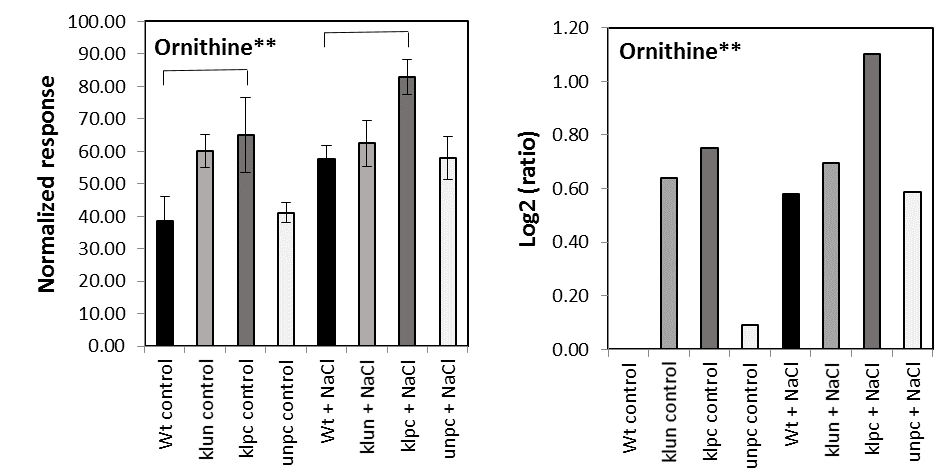


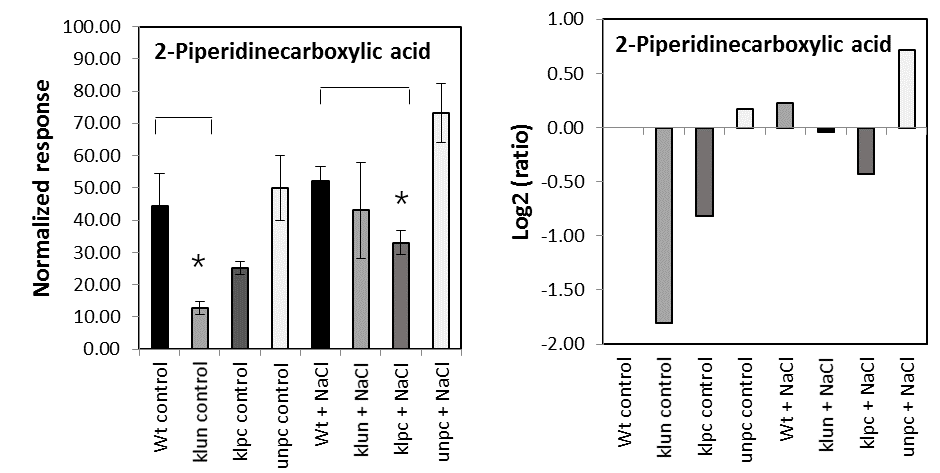


**Fig. S5. Exemplary metabolites in *klun*, *klpc* and *unpc* mutant roots compared to Wt under salt stressed and non-stressed control conditions**. Normalized responses and log_2_-transformed response ratios were relative to Wt control. Bar diagrams show medians and standard errors. Stars indicate significant changes compared to Wt control (*P*<0.05, t-test).

**Table S1. *F* values and significance levels for two-way ANOVA of the data for fresh weight and length of flower stem of different genotypes plants (Wt, *klpc*, *klun*, *unpc*, *hkt1*) under Na (0 and 100 mM) treatment.**

| **Source** | **Treatment** | | **Genotype** | | **Treatment × Genotype** | |
| --- | --- | --- | --- | --- | --- | --- |
|  | **Exp 1** | **Exp 2** | **Exp 1** | **Exp 2** | **Exp 1** | **Exp 2** |
| Fresh weight of rosette leave | 119.37 *** | 315.99*** | 7.47*** | 6.24*** | 0.53 n.s. | 2.28 n.s. |
| Fresh weight of inflorescence | 36.76*** | 44.27*** | 9.68*** | 13.20*** | 0.80 n.s. | 3.92 n.s. |
| Length of flower stem | 22.92*** | 117.21*** | 27.78*** | 133.41*** | 0.42 n.s. | 22.25 ** |

*** P<0.001, **P<0.01, *P<0.05., n.s. = non-significant differences.

**Table S2. *F* values and significance levels for two-way ANOVA of the data for Na content and K content in different genotypes plants (Wt, *klpc*, *klun*, *unpc*, *hkt1*) under Na (0 and 100 mM) treatment.**

| **Source** | **Treatment** | | **Genotype** | | **Treatment × Genotype** | |
| --- | --- | --- | --- | --- | --- | --- |
|  | **Exp 1** | **Exp 2** | **Exp 1** | **Exp 2** | **Exp 1** | **Exp 2** |
| Na Content of rosette leave | 733.00*** | 1037.55*** | 316.87*** | 592.16*** | 117.32*** | 191.59*** |
| Na Content of inflorescence | 575.96*** | 241.52*** | 129.35*** | 53.75*** | 65.67 n.s. | 26.80 n.s. |
| K Content of rosette leave | 172.79*** | 141.19*** | 39.02*** | 33.84*** | 1.31*** | 2.43*** |
| K Content of inflorescence | 42.13*** | 62.76*** | 4.30 * | 0.76 n.s | 1.52 n.s. | 0.63 n.s. |

*** P<0.001, **P<0.01, *P<0.05., n.s. = non-significant differences.

**Table S3. *F* values and significance levels for two-way ANOVA of the data for Na^+^ : K^+^ ratio in different genotypes plants (Wt, *klpc*, *klun*, *unpc*, *hkt1*) under Na (0 and 100 mM) treatment.**

| **Source** | **Treatment** | | **Genotype** | | **Treatment × Genotype** | |
| --- | --- | --- | --- | --- | --- | --- |
|  | **Exp 1** | **Exp 2** | **Exp 1** | **Exp 2** | **Exp 1** | **Exp 2** |
| Na^+^ : K^+^ ratio of rosette leave | 120.37*** | 141.21*** | 20.07*** | 13.02*** | 1.32 n.s | 4.93 ** |
| Na^+^ : K^+^ ratio of inflorescence | 79.05*** | 95.68*** | 20.43** | 11.85*** | 0.93 n.s | 1.78 n.s |

*** P<0.001, **P<0.01, *P<0.05., n.s. = non-significant differences.
